# Tripartite evolutionary game analysis of medical data governance: interactions among government, medical institutions, and third-party assessment agencies

**DOI:** 10.3389/fpubh.2026.1862621

**Published:** 2026-07-17

**Authors:** Xingxian Liu, Jing Gong, Jusheng Liu

**Affiliations:** School of Economics and Management, Shanghai University of Political Science and Law, Shanghai, China

**Keywords:** collusive behavior, evolutionary game, government regulation, medical data management, replicated dynamic equation

## Abstract

In the era of the digital economy, medical data has emerged as a core production factor, yet revitalizing its value and achieving effective governance remain critical challenges. Based on evolutionary game theory, this paper constructs a three-party game model encompassing the government, medical institutions, and third-party assessment agencies. The study reveals that the system's evolutionary outcome is highly sensitive to initial parameter configurations, with strategic interdependencies among the three stakeholders. Specifically, government regulatory intensity, incentive-punishment mechanisms, and the cost-benefit structures of medical institutions and third-party assessment agencies constitute pivotal determinants of system stability. To facilitate medical data circulation and achieve effective governance, policymakers should increase incentives for truthful data provision by medical institutions, impose penalties against collusive behavior, strengthen incentives and penalties for third-party assessment agencies, and enhance benefits for medical institutions that provide truthful data. This study contributes to expanding the application of evolutionary game theory in medical data governance while offering actionable policy implications.

## 1 Introduction

In today's information age, data has become a fundamental factor of production, playing a key role in driving industrial innovation and technological progress (1). Unlike general commercial data, medical data has a unique dual attribute: it has high clinical value for precision medicine and artificial intelligence model development (2). With the rapid rise of the digital economy, medical institutions have accumulated a large amount of clinical, imaging, and genomic data. However, how to transform these static resources into sustainable data assets that can circulate, add value, and regenerate value has become a core bottleneck for the government's digital transformation of healthcare (3). This process involves complex strategic interactions among multiple stakeholders (medical institutions, government, and third-party assessment agencies), where the conflict between value release and risk control continues to challenge the establishment of sustainable operational mechanisms (4).

In real life, medical data management involves multiple stakeholders with conflicting interests and limited rationality. Medical institutions have private information about data quality, and when faced with buyer price suppression, they often tend to falsify, conceal, or collude with third parties. Third-party assessment agencies face the moral hazard of balancing high cost accurate inspection and low-cost evasion or false reporting, leading to collusion between both parties in the form of low-quality data and false certification, squeezing high-quality data out of circulation and causing data supply shortages and systemic risks for medical artificial intelligence research and public health decision-making. As the maximization of social welfare and ultimate responsibility bearer, when data breaches or fraudulent sharing occur, the government bears serious reputation costs. The government attempts to break this collusion equilibrium by acting as a provider of market infrastructure and designing incentive compatible mechanisms that subsidize high-quality data in real circulation to correct positive externalities that the market has not internalized, as well as conducting random checks to punish fraudulent behavior and prevent privacy breaches as a deterrent mechanism. Therefore, how to construct a game model around medical data governance, analyze the interactive relationship between the government, medical institutions, and third-party assessment agencies, has good theoretical and practical significance for clarifying the mechanism of medical data governance.

Although existing literature has explored medical data circulation from technical perspectives, such as blockchain-based data sharing and traceability, the persistent governance failure of medical data quality remains inadequately explained. The fundamental barrier is not technical but lies in the misalignment of interests among stakeholders: the government faces information asymmetry in regulation; medical institutions may be incentivized to falsify data to maximize short-term returns; and third-party assessment agencies may collude with medical institutions to provide fraudulent evaluations. These interactions are inherently strategic: each party's optimal decision depends on the anticipated choices of the others. A game-theoretic framework is therefore theoretically necessary to explain why and under what conditions these parties can achieve coordination or fall into governance failure.

Furthermore, classic game theory assumes fully rational agents with complete information, which does not reflect the real-world behavior of stakeholders in medical data governance, who typically operate under bounded rationality, incomplete information, and adaptive learning. Evolutionary game theory relaxes the full-rationality assumption and models strategy dynamics through replicator dynamics, capturing the gradual adjustment of strategies over time as players imitate more successful strategies. This is particularly suitable for describing the dynamic, path-dependent process by which all three parties revise their strategies in response to changing regulatory and market conditions. Static game models, by contrast, cannot capture this evolving interaction process.

Based on this, this study considers cost, reputation, reward and punishment mechanisms, and reputation mechanisms. By constructing a tripartite evolutionary game model between medical institutions, third-party assessment agencies, and the government, it aims to address the multiple challenges of low quality medical data governance and collusion behavior. The innovations and contributions of this paper are outlined as follows. First, this article analyzes for the first time the game relationship among medical institutions, governments, and third-party assessment agencies in medical data governance, especially considering the collusion behavior between medical institutions and third-party assessment agencies. This provides meaningful reference and inspiration for medical data governance. Second, by employing evolutionary game theory, this study develops a novel model for medical data circulation and management that effectively elucidates the interactions among medical institutions, governments, and third-party assessment agencies. Third, this study provides practical guidance for current medical data circulation practices and presents governance strategies to enhance medical data transactions and regulation.

The structure of this paper is organized as follows: Section 1 introduces the background of this study and presents the research questions. Literature review is presented in Section 2. Section 3 presents the model formulation and assumptions. The analysis of the evolutionary game model is detailed in Section 4. Numerical simulations are discussed in Section 5, while Section 6 provides the discussion and conclusion.

## 2 Literature review

### 2.1 Medical data governance

Medical data, as an important asset, has significant value (5, 6). However, for a long time, medical data has not been able to effectively realize its value, which can be attributed to various reasons. On the one hand, some medical data may have quality issues that prevent it from being circulated in the market, such as incomplete data, inaccurate data, redundant data, missing document records, insufficient data discoverability, and lack of ethical considerations (7); On the other hand, the circulation of data also involves multiple stakeholders, and there is no well-established mechanism for distributing benefits, resulting in many problems in data governance. In terms of medical data governance, existing research has explored it from different perspectives. Among them, some studies have explored how to governance medical data and how to leverage its value from a management perspective. For example, Donia et al. (8) considered that in order for data to be detached from the original production context and transformed into tradable assets, it must rely on agency work, that is, by arranging specific people, infrastructure, and material equipment to enable data to represent or replace other things. Therefore, data needs to be governed, and this process also involves multiple parties, such as governments, data owners, third-party assessment agencies, etc. Paparova et al. (9) interpreted the data governance space and found that the data governance space does not refer to the internal system of an organization, but rather to the dynamically changing governance relationship field formed when data flows across subjects. Yao and Liu (10) explored the interactive relationship between medical data in medical data management institutions, medical data operation departments, and data related entities, and considered the important roles of privacy, security, and ethical risks in data governance. Besides, some research explored the value co-creation among multiple stakeholders (11) and law problems in medical data trade (12).

In addition, some studies have explored how to medical data governance from a technical perspective. For example, privacy preserving computation (13), blockchain-based rights confirmation (14, 15), and static institutional designs (e.g., legal regulations and ethical frameworks) (16–19), both emphasizing external regulation to ensure data security during the process of data governance. Specifically, Zhang et al. (20) reviewed AI applications in gerontology, focusing on multi-modal data analysis including electronic health records, genomic data, medical imaging, and wearable device metrics. Meanwhile, Jan and Sofi (21) explored medical Internet of Things (IoT) innovations for optimizing data processing and management resources. Eden et al. (22) proposed a systematic federated learning governance mechanism framework based on the sovereignty, ethics, privacy, and risk of harm of medical data, which enables the release of data value while protecting privacy.

### 2.2 Evolutionary game applications in data governance

In data governance, data, as an important asset, has certain transactional and value attributes. How to trade and evaluate the value of data constrains the interests of all parties. Therefore, building a harmonious sharing mechanism is particularly important for all parties. In recent years, with the continuous development and expansion of the application scope of evolutionary game theory (23–26), scholars have used evolutionary game theory to explore data governance and achieved good results. For instance, Fu et al. (27) developed a tripartite game model to analyze the dynamic interactions and evolutionary stability among government entities, data developers, and data users within value co-creation networks throughout the data management process. Li et al. (28) constructed a game model to explore data trading behaviors among data providers, users, and regulators. Benko et al. (29) utilized game theory to illustrate the social dilemmas faced by managers regarding open data. Chen et al. (30) integrated incentives based on reputation and payment, employing smart contracts and evolutionary game theory to investigate data sharing. Wang et al. (31) explored the interactive relationship between grassroots governments, local governments, and third-party regulatory agencies in the process of data governance, and found that dynamic reward and punishment mechanisms and increased rent-seeking costs can accelerate the convergence of all parties to a stable regulatory equilibrium.

In addition, some studies have explored medical data governance using evolutionary game theory. For example, Wang et al. (32) explored the interactive relationship between patients, government, and data users based on the use of medical data, and found that patient participation and government regulatory efficiency are important factors affecting system stability. Gao et al. (33) explored the interactive relationship between data providers, medical data sharing platforms, and data demanders in the process of medical data governance, and found that platforms and regulatory agencies should regulate and govern data flow quality through reward and punishment mechanisms. Tian and Chen (34) developed a tripartite evolutionary game model encompassing government, hospitals, and patients to investigate the governance and circulation mechanisms of electronic medical records. Their findings demonstrate that patient supervision serves as a positive external oversight force, and an appropriately structured incentive mechanism can effectively motivate hospitals to engage proactively. Zhang et al. (35) explored the strategic interaction between comprehensive hospitals, community health service centers, and government agencies around medical knowledge sharing. The study found that increasing government incentives and punishments, while strengthening patient feedback mechanisms, can effectively promote medical knowledge sharing. Wang et al. (36) explored the strategic interaction between patients, platforms, and users around the use of medical data, and found that privacy and security protection, as well as the benefits of data sharing, are important factors that encourage patient participation in sharing.

In summary, it can be seen that existing research has explored the governance of medical data from both management and technology perspectives. Besides, there are also studies that explore medical data governance from a dynamic perspective using evolutionary game theory, providing valuable insights for data flow and governance. However, there are still few discussions on the governance of medical data based on dynamic perspectives, especially regarding the governance of medical data by medical institutions, governments, and third-party assessment agencies. In real life, the third-party assessment agencies play an important role in the medical data governance, it can make sure the value of data and can help the data to facilitate the pricing and circulation of data. If there is no consider the role of third-party assessment agencies, it will obstacle to understanding data circulation and achieving value co creation of medical data. Therefore, this study will explore the interactive game relationship among the three parties in data governance to achieve good governance of medical data, providing theoretical and practical inspiration for multi-party value co creation.

## 3 Model formulation and assumptions

### 3.1 Model formulation

Different from the entirely rational characteristics of participants in traditional game theory, evolutionary game theory posits that players are boundedly rational. Participants must undergo an adaptive adjustment process to attain optimal strategies. In the context of medical data management, the government, which pursues social public interests, medical institutions that seek economic benefits, and third-party assessment agencies all function as boundedly rational participants. They dynamically adjust their strategies by observing and comparing the interests of all stakeholders. Consequently, evolutionary game theory is well suited for examining medical data management issues centered on value co-creation.

Medical data holds significant value as an asset. Medical institutions, as custodians of this data, face three strategic options: disclosing accurate information to enhance value, withholding data due to concerns about operational efficiency, or providing falsified data to inflate asset value. To encourage data sharing, the government establishes policies to regulate both medical institutions and third-party assessment agencies, implementing reward-penalty mechanisms based on their behavior. However, the government faces its own strategic tradeoff between stringent regulation (requiring substantial enforcement resources) and weak regulation (risking quality compromise).

**Figure 1:**
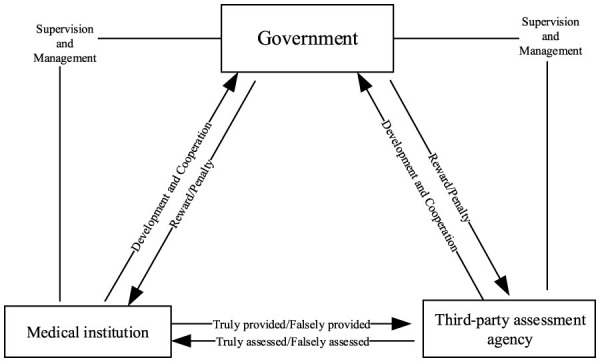
Game model among stakeholders in medical data governance.

Third-party assessment agencies, acting as data evaluators, can conduct either accurate assessments or misleading evaluations, and may collude with medical institutions driven by mutual interests. Thus, medical data circulation involves a tripartite game among medical institutions, assessment agencies, and the government. The dynamics of their interactions are illustrated in Figure 1.

### 3.2 Assumptions

Based on the theoretical analysis presented above, this study proposes the following assumptions:

**Assumption 1**. In the context of data governance, various stakeholders including the government, medical institution, and third-party assessment agencies participate in a complex interplay characterized by bounded rationality. The government faces two strategic options: strong regulation and weak regulation. The probability of implementing strong regulation is denoted as *x*, while the probability of weak regulation is represented as 1 − *x*. When the government opts for strong regulation, it incurs higher costs, represented as *C*_*i*_; conversely, the costs associated with weak regulation are denoted as *C*_*j*_. Here, in fact, in addition to regulatory costs, strong regulation refers to regulation with high enforcement intensity. This is manifested in regulatory agencies investing sufficient regulatory resources, conducting special inspections, and tracing the entire process. In this way, high investment and high-intensity regulation can discover false behavior of medical institutions and third-party evaluation agencies; Compared to strong regulation, weak regulation is a low-frequency and low-cost random inspection that often fails to truly detect violations, resulting in lower regulatory costs. Under weak regulation, due to the government's limited investment, it is often difficult to detect fraudulent activities by medical institutions and third-party assessment agencies. Under government regulation, if medical institutions provide truthful data and third-party assessment agencies conduct truthful assessments, the government's reputation is enhanced by an amount *E*. Conversely, if medical institutions and third-party assessment agencies collude to defraud the government of incentives and subsidies, this can result in a decrease in the government's reputation by an amount *F*.

**Assumption 2**. The medical institution's strategic options consist of true provision and false provision. The probability of the medical institutions providing truthful data is denoted as *y*, while the probability of supplying false data is represented as 1 − *y*. When the medical institution provides truthful data, it generates a revenue of *R*_*i*_ but also faces a data leakage risk quantified as *C*_*n*_. Conversely, providing false data results in a revenue of *M*. Due to government regulations, under strong regulatory conditions, if the medical institution opts for false provision, it will incur a penalty of *S*_*j*_. In contrast, under weak government regulation, if the medical institution chooses truthful provision, it will receive a reward of *A*_*j*_.

**Assumption 3**. The strategic choices of third-party assessment agencies include truthful assessment and false assessment. The probability of a truthful assessment is denoted as *z*, while the probability of a false assessment is represented as 1 − *z*. The cost associated with a true assessment conducted by a third-party agency is *C*_*m*_, whereas the cost for a false assessment is *C*_*x*_, *C*_*m*_>*C*_*x*_. When a third-party agency performs a truthful assessment, it generates revenue of *R*_*j*_, while revenue from false assessment data amounts to *N*. Due to government regulations, agencies face a penalty of *S*_*i*_ for conducting false assessments under stringent regulations. Conversely, under weak government regulations, agencies may receive a reward of *A*_*i*_ for opting for truthful assessments. In practice, the third-party assessment agencies may obtain illicit gains through collusion with medical institutions (e.g., bribes for inflated quality reports), making false assessments temporarily profitable under weak regulatory oversight.

**Assumption 4**. In the process of data assessment, there may be collusion between medical institutions that provide false data to third-party assessment agencies for fraudulent evaluations. In such cases, if the government enforces strong regulation, the collusion between medical institutions and agencies may incur a penalty, denoted as *P*. Here, unlike unilateral false behavior, this collusion behavior formed by false information from both parties often poses greater harm to society. Therefore, the punishment will become more severe. To maintain generality, we specifically use *P* to represent the government's punishment for collusion behavior between medical institutions and third-party assessment agencies. To facilitate this collusion, medical institutions will transfer certain benefits, referred to as *B*, to the third-party assessment agencies, while also receiving additional benefits, labeled *R*_*m*_. Under normal circumstances, if a medical institution opts to provide false data, the net income (gross revenue minus compliance costs) may exceed that of truthful data provision strategy when regulation probability of government is sufficiently low.

The symbols and descriptions of the parameters utilized in the assumptions outlined above are presented in Table 1.

**Table 1:** Notations of the parameters in the payoff matrix.

Symbol	Description
*C_*i*_*	The cost of government implementing strong regulations
*C_*j*_*	The cost of government implementing weak regulations
*C_*m*_*	The cost of obtaining truthful assessment data from third-party assessment agencies
*C_*n*_*	Risk of data leakage from medical institutions providing truthful data
*C_*x*_*	The cost of obtaining falsely assessment data from third-party assessment agencies
*E*	The increase in government reputation
*F*	The decrease in government reputation
*M*	Profits gained by medical institutions through providing false data
*N*	Profits gained by third-party assessment agencies through falsely evaluating data
*A_*i*_*	Government incentives awarded to third-party assessment agencies for truthfully assessed data under weak government oversight
*A_*j*_*	Government incentives awarded to medical institutions for truthfully providing data under weak government oversight
*R_*i*_*	Benefits for medical institutions when providing truthful data
*R_*j*_*	Benefits for third-party assessment agencies when choosing truthful assessment data
*R_*m*_*	Benefits obtained through collusion between medical institutions and agencies
*S_*i*_*	Penalties imposed on third-party assessment agencies for false activities under strong government regulation
*S_*j*_*	Penalties imposed on medical institutions for false activities under strong government regulation
*P*	Penalties that should be imposed on third-party assessment agencies and medical institutions for collusion under strong regulation
*B*	Collusion costs paid by medical institutions to third-party assessment agencies

### 3.3 Payoff matrix construction

Based on the aforementioned assumptions, we can identify the government's strategic options as strong regulation and weak regulation, the medical institutions' strategic options as truthful provision and false provision, and the third-party assessment agency's strategic options as truthful assessment and false assessment. Consequently, there are eight possible strategy combinations in the tripartite evolutionary game involving the government, medical institutions, and the third-party assessment agency. The payoff matrix for each strategy is presented in Table 2.

**Table 2:** The payoff matrix for the game.

Number	Strategy selection	Government	Enterprise	Third-party assessment agency
①	(Strong regulation, truthful provision, truthful assessment)	*E* − *C*_*i*_	*R*_*i*_ − *C*_*n*_	*R*_*j*_ − *C*_*m*_
②	(Strong regulation, false provision, truthful assessment)	*S*_*j*_ − *C*_*i*_	*M* − *S*_*j*_	*R*_*j*_ − *C*_*m*_
③	(Strong regulation, false provision, false assessment)	2*P* − *C*_*i*_	*R*_*m*_ − *B* − *P*	*B* − *C*_*x*_ − *P*
④	(Strong regulation, truthful provision, false assessment)	*S*_*i*_ − *C*_*i*_	*R*_*i*_ − *C*_*n*_	*N* − *C*_*x*_ − *S*_*i*_
⑤	(Weak regulation, truthful provision, truthful assessment)	− *C*_*j*_ − *A*_*i*_ − *A*_*j*_	*R*_*i*_ + *A*_*j*_ − *C*_*n*_	*A*_*i*_ + *R*_*j*_ − *C*_*m*_
⑥	(Weak regulation, false provision, truthful assessment)	-*C*_*j*_	*M*	*A*_*i*_ + *R*_*j*_ − *C*_*m*_
⑦	(Weak regulation, false provision, false assessment)	− *C*_*j*_ − *F*	*R*_*m*_ − *B*	*B* − *C*_*x*_
⑧	(Weak regulation, truthful provision, false assessment)	-*C*_*j*_	*R*_*i*_ + *A*_*j*_ − *C*_*n*_	*N* − *C*_*x*_

### 3.4 Game model construction

According to evolutionary game theory and the payoff matrix presented in Table 2, the replication dynamic equations for the government, medical institutions, and third-party assessment agencies are calculated as follows. Assume that the expected returns of the government adopting strong regulation and weak regulation strategies are *E*_11_ and *E*_12_, respectively, while the average expected return is *E*_1_.


$$\begin{array}{l} E_{11}=yz\left(E-S_{j}+2P-S_{i}\right)+z\left(S_{j}-2P\right)\\ \;-y\left(2P-S_{i}\right)+2P-C_{i} \end{array}$$ (1)

$$\begin{array}{l} E_{12}=-yz\left(A_{i}+A_{j}+F\right)+\left(y+z-1\right)F-C_{j} \end{array}$$ (2)

$$\begin{array}{l} E_{1}=\;xE_{11}+\left(1-x\right)E_{12} \end{array}$$ (3)

The dynamic replication equation of the government is represented as *F*(*x*).


$$\begin{array}{l} F(x)=x(1-x)[yz\left(E+F-S_{j}+2P-S_{i}+A_{i}+A_{j}\right)\\ \;-y\left(2P-S_{i}\right)+z\left(S_{j}-2P\right)+2P-C_{i}\\ \;-\left(y+z-1\right)F+C_{j} \end{array}$$ (4)

Assuming that the expected returns of the medical institution adopting the truthful provision strategy and the false provision strategy are *E*_21_ and *E*_22_ respectively, the average expected return is *E*_2_.


$$\begin{array}{l} E_{21}=R_{i}-C_{n}+\left(1-x\right)A_{j} \end{array}$$ (5)

$$\begin{array}{l} E_{22}=zM+\left(1-z\right)\left(R_{m}-B\right)-xzS_{j}-x\left(1-z\right)P \end{array}$$ (6)

$$\begin{array}{l} E_{2}=yE_{21}+\left(1-y\right)E_{22} \end{array}$$ (7)

The replication dynamic equation of the medical institution is represented as *F*(*y*).


$$\begin{array}{l} F(y)=y(1-y)[R_{i}-C_{n}-R_{m}+B+A_{j}+z(R_{m}-B-M)\\ \;+x(zS_{j}-A_{j}+P-zP)] \end{array}$$ (8)

Assuming the expected returns of the third-party assessment agency employing the truthful assessment strategy and the false assessment strategy are *E*_31_ and *E*_32_, respectively, the average expected return is *E*_3_.


$$\begin{array}{l} E_{31}=x\left(R_{j}-C_{m}\right)+\left(1+x\right)\left(A_{i}+R_{j}-C_{m}\right) \end{array}$$ (9)

$$\begin{array}{l} E_{32}=yN+\left(1-y\right)B-xyS_{i}-x\left(1-y\right)P-C_{x} \end{array}$$ (10)

$$\begin{array}{l} E_{3}=zE_{31}+\left(1-z\right)E_{32} \end{array}$$ (11)

The replication dynamic equation of the third-party assessment agency is represented as *F*(*z*).


$$\begin{array}{l} F(z)=z(1-z)[x(R_{j}-C_{m})+(1+x)(A_{i}+R_{j}-C_{m})\\ \;-(yN+(1-y)B-xyS_{i}-x(1-y)P-C_{x})] \end{array}$$ (12)

### 3.5 Three-party evolutionary game theory model stability analysis

Since the government, medical institutions, and third-party assessment agencies have limited rationality, the optimal strategy choice cannot be achieved in a single decision. Therefore, for stability analysis, we refer to Friedman's method of local stability analysis using the Jacobian matrix of dynamic equations (37). Three replicator dynamic equations are combined to form a replicator dynamic system. The solution to this system of equations represents the equilibrium solution of the evolutionary game theory model. The replicator dynamic systems for the government, medical institutions, and third-party assessment agencies are presented as follows.


$$\begin{array}{l} F(x)=x(1-x)[yz(E+F-S_{j}+2P-S_{i}+A_{i}+A_{j})\\ \;-y(2P-S_{i})+z(S_{j}-2P)+2P-C_{i}-(y+z-1)F\\ \;+C_{j}] \end{array}$$ (13)

$$\begin{array}{l} F(y)=y(1-y)[R_{i}-C_{n}-R_{m}+B+A_{j}+z(R_{m}-B-M)\\ \;+x(zS_{j}-A_{j}+P-zP)] \end{array}$$ (14)

$$\begin{array}{l} F(z)=z(1-z)[x(R_{j}-C_{m})+(1+x)(A_{i}+R_{j}-C_{m})\\ \;-(yN+(1-y)B-xyS_{i}-x(1-y)P-C_{x})] \end{array}$$ (15)

Thus, the Jacobian matrix of the three-party evolutionary game system for medical data management is presented below.


$$\begin{array}{l} J=\left[\begin{array}{ccc} J_{11} & J_{12} & J_{13}\\ J_{21} & J_{22} & J_{23}\\ J_{31} & J_{32} & J_{33} \end{array}\right]=\left[\begin{array}{ccc} \frac{\partial F\left(x\right)}{\partial x} & \frac{\partial F\left(x\right)}{\partial y} & \frac{\partial F\left(x\right)}{\partial z}\\ \frac{\partial F\left(y\right)}{\partial x} & \frac{\partial F\left(y\right)}{\partial y} & \frac{\partial F\left(y\right)}{\partial z}\\ \frac{\partial F\left(z\right)}{\partial x} & \frac{\partial F\left(z\right)}{\partial y} & \frac{\partial F\left(z\right)}{\partial z} \end{array}\right] \end{array}$$ (16)

*F*(*x*), *F*(*y*), and *F*(*z*) are partially differentiated with respect to *x*, *y*, and *z*, respectively, where:


$$\begin{array}{l} J_{11}=(1-2x)[yz(E+F-S_{j}+2P-S_{i}+A_{i}+A_{j})-y(2P-S_{i})\\ \;+z(S_{j}-2P)+2P-C_{i}-(y+z-1)F+C_{j}] \end{array}$$ (17)

$$\begin{array}{l} J_{12}=x(1-x)[z(E+F-S_{j}+2P-S_{i}+A_{i}+A_{j})\\ \;-(2P-S_{i})-F] \end{array}$$ (18)

$$\begin{array}{l} J_{13}=x(1-x)[y(E+F-S_{j}+2P-S_{i}+A_{i}+A_{j})\\ \;+(S_{j}-2P)-F] \end{array}$$ (19)

$$\begin{array}{l} J_{21}=y\left(1-y\right)\left[zS_{j}+\left(1-z\right)P-A_{j}\right] \end{array}$$ (20)

$$\begin{array}{l} J_{22}=(1-2y)[R_{i}-C_{n}-R_{m}+B+A_{j}+z(R_{m}-B-M)\\ \;+x(zS_{j}-A_{j}+P-zP)] \end{array}$$ (21)

$$\begin{array}{l} J_{23}=y\left(1-y\right)\left[x\left(S_{j}-p\right)+R_{m}-B-M\right] \end{array}$$ (22)

$$\begin{array}{l} J_{31}=z\left(1-z\right)\left[2R_{j}+A_{i}-2C_{m}+P+y\left(S_{i}-P\right)\right] \end{array}$$ (23)

$$\begin{array}{l} J_{32}=z\left(1-z\right)\left[B+x\left(S_{i}-P\right)-N\right] \end{array}$$ (24)

$$\begin{array}{l} J_{33}=(1-2z)[x(R_{j}-C_{m})+(1+x)(A_{i}+R_{j}-C_{m})\\ \;-(yN+(1-y)B-xyS_{i}-x(1-y)P-C_{x})] \end{array}$$ (25)

An evolutionary stable strategy (ESS) refers to a process in which individuals participating in a game within a population continuously adjust their strategies to maximize the interests of the majority, thereby achieving a dynamic equilibrium. Given the equations *F*(*x*) = 0, *F*(*y*) = 0, and *F*(*z*) = 0, we can identify 8 equilibrium points of the system: *E*_1_(0, 0, 0), *E*_2_(1, 0, 0), *E*_3_(0, 1, 0), *E*_4_(0, 0, 1), *E*_5_(1, 1, 0), *E*_6_(1, 0, 1), *E*_7_(0, 1, 1), and *E*_8_(1, 1, 1).

## 4 Evolutionary game model analysis

According to the Lyapunov stability theorem (38), the stability of equilibrium points can be determined by analyzing the eigenvalues of the Jacobian matrix. If all eigenvalues (λ) of the equilibrium point are positive, it is classified as an unstable point. If the eigenvalues (λ) of the equilibrium point include both positive and negative values, it is considered a saddle point. Conversely, if all eigenvalues (λ) of the equilibrium point are negative, it is deemed a stable point. The eigenvalues for each equilibrium point of the tripartite evolutionary game system in medical data management are presented in Table 3.

**Table 3:** Eigenvalues corresponding to the system equilibrium point.

Stable equilibriums	λ_1_	λ_2_	λ_3_
*E*_1_(0, 0, 0)	*C*_*j*_ − *C*_*i*_ + *F* + 2*P*	*R*_*i*_ − *C*_*n*_ − *R*_*m*_ + *B* + *A*_*j*_	*A*_*i*_ + *R*_*j*_ − *C*_*m*_ − *B* + *C*_*x*_
*E*_2_(1, 0, 0)	*C*_*i*_ − *C*_*j*_ − *F* − 2*P*	*R*_*i*_ − *C*_*n*_ − *R*_*m*_ + *B* + *P*	2*A*_*i*_ + 3*R*_*j*_ − 3*C*_*m*_ − *B* + *C*_*x*_ + *P*
*E*_3_(0, 1, 0)	*C*_*j*_ − *C*_*i*_ + *S*_*i*_	− (*R*_*i*_ − *C*_*n*_ − *R*_*m*_ + *B* + *A*_*j*_)	*A*_*i*_ + *R*_*j*_ − *C*_*m*_ − *N* + *C*_*x*_
*E*_4_(0, 0, 1)	*C*_*j*_ − *C*_*i*_ + *S*_*j*_	*A*_*j*_ − *C*_*n*_ − *M* + *R*_*i*_	− (*A*_*i*_ + *R*_*j*_ − *C*_*m*_ − *B* + *C*_*x*_)
*E*_5_(1, 1, 0)	*C*_*i*_ − *C*_*j*_ − *S*_*i*_	− (*R*_*i*_ − *C*_*n*_ − *R*_*m*_ + *B* + *P*)	2*A*_*i*_ + 3*R*_*j*_ − 3*C*_*m*_ − *N* + *C*_*x*_ + *S*_*i*_
*E*_6_(1, 0, 1)	*C*_*i*_ − *C*_*j*_ − *S*_*j*_	*R*_*i*_ − *C*_*n*_ − *M* + *S*_*j*_	− (2*A*_*i*_ + 3*R*_*j*_ − 3*C*_*m*_ − *B* + *C*_*x*_ + *P*)
*E*_7_(0, 1, 1)	*A*_*i*_ + *A*_*j*_ + *C*_*j*_ − *C*_*i*_ + *E*	*C*_*n*_ + *M* − *R*_*i*_ − *A*_*j*_	− (*A*_*i*_ + *R*_*j*_ − *C*_*m*_ − *N* + *C*_*x*_)
*E*_8_(1, 1, 1)	*C*_*i*_ − *C*_*j*_ − *E* − *A*_*i*_ − *A*_*j*_	*C*_*n*_ + *M* − *R*_*i*_ − *S*_*j*_	− (2*A*_*i*_ + 3*R*_*j*_ − 3*C*_*m*_ − *N* + *C*_*x*_ + *S*_*i*_)

According to evolutionary game theory, the equilibrium states of the system described above can be classified into four distinct scenarios.

### 4.1 Scenario 1

When *C*_*i*_ − *C*_*j*_ − *E* − *A*_*i*_ − *A*_*j*_ < 0, *C*_*n*_ + *M* − *R*_*i*_ − *S*_*j*_ < 0, and 2*A*_*i*_ + 3*R*_*j*_ − 3*C*_*m*_ − *N* + *C*_*x*_ + *S*_*i*_>0, we can conclude that *C*_*i*_<*C*_*j*_ + *E* + *A*_*i*_ + *A*_*j*_ and *M* − *S*_*j*_<*R*_*i*_ − *C*_*n*_. In this scenario, the reputation benefits brought by the government's choice of strong regulatory strategies far outweigh the costs it incurs. Consequently, the government's strategy will shift from weak to strong regulation. From the perspective of medical institutions' strategic choices, the benefits of providing false data are less than those of providing truthful data, leading medical institutions to transition from false provision to truthful provision. Additionally, when third-party assessment agencies determine that the benefits of truthful assessment surpass those of false assessment, their strategy will shift from false to truthful assessment. The stability analysis of the equilibrium is presented in Table 4.

**Table 4:** System equilibrium point stability in scenario 1.

Equilibrium point	Eigenvalue			Stability
	λ_1_	λ_2_	λ_3_	
*E*_1_(0, 0, 0)	>0	>0	>0	Unstable
*E*_2_(1, 0, 0)	< 0	>0	Uncertain	Saddle point
*E*_3_(0, 1, 0)	< 0	< 0	>0	Saddle point
*E*_4_(0, 0, 1)	< 0	>0	< 0	Saddle point
*E*_5_(1, 1, 0)	>0	< 0	Uncertain	Saddle point
*E*_6_(1, 0, 1)	< 0	>0	Uncertain	Saddle point
*E*_7_(0, 1, 1)	>0	< 0	< 0	Saddle point
*E*_8_(1, 1, 1)	< 0	< 0	< 0	ESS

**Figure 2:**
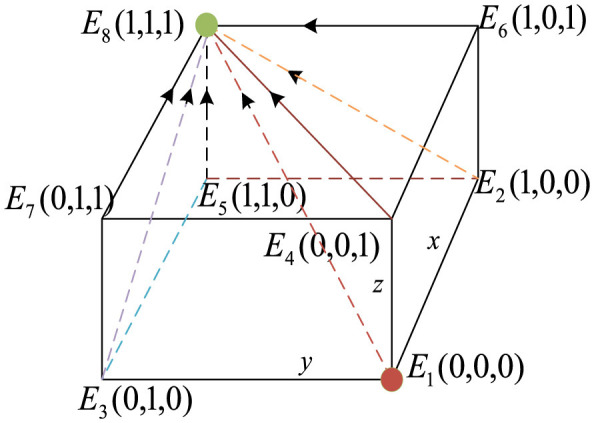
Three-party game phase diagram under scenario 1.

In Table 4, according to the Lyapunov stability theorem (38), the point *E*_1_(0, 0, 0) represents an unstable equilibrium state, while *E*_8_(1, 1, 1) signifies a stable equilibrium state. The strategy associated with a stable equilibrium is known as the evolutionarily stable strategy. Consequently, the evolutionary trajectory of the system will transition from *E*_1_ to *E*_8_. In this context, if the government implements a regulatory strategy and enforces strict penalties, medical institutions may incur significant fines for submitting false data, prompting them to provide truthful information. Similarly, third-party assessment agencies will also opt for truthful assessments due to the high penalties involved, resulting in a shift of the strategy toward *E*_8_(1, 1, 1). The phase diagram illustrating this condition is presented in Figure 2.

### 4.2 Scenario 2

When *C*_*i*_ − *C*_*j*_ − *S*_*j*_ < 0, *R*_*i*_ − *C*_*n*_ − *M* + *S*_*j*_ < 0, 2*A*_*i*_ + 3*R*_*j*_ − 3*C*_*m*_ − *B* + *C*_*x*_ + *P*>0, the *E*_6_(1, 0, 1) is the evolutionary stable strategy. In this scenario, the cost to the government of implementing strong regulation is lower than the overall cost of adopting weak regulation, prompting a shift in the government's strategy from weak to strong regulation. For medical institutions, the advantages of providing false data outweigh the benefits of submitting truthful data, leading them to transition from truthful provision to false provision. Meanwhile, third-party assessment agencies find that the benefits of delivering truthful assessments surpass those of providing false assessments, resulting in a shift from false to truthful assessments. The stability analysis of the equilibrium is presented in Table 5.

**Table 5:** System equilibrium point stability in scenario 2.

Equilibrium point	Eigenvalue			Stability
	λ_1_	λ_2_	λ_3_	
*E*_1_(0, 0, 0)	>0	< 0	>0	Saddle point
*E*_2_(1, 0, 0)	< 0	< 0	>0	Saddle point
*E*_3_(0, 1, 0)	>0	>0	>0	Unstable
*E*_4_(0, 0, 1)	>0	< 0	< 0	Saddle point
*E*_5_(1, 1, 0)	< 0	>0	>0	Saddle point
*E*_6_(1, 0, 1)	< 0	< 0	< 0	ESS
*E*_7_(0, 1, 1)	>0	>0	< 0	Saddle point
*E*_8_(1, 1, 1)	< 0	>0	< 0	Saddle point

**Figure 3:**
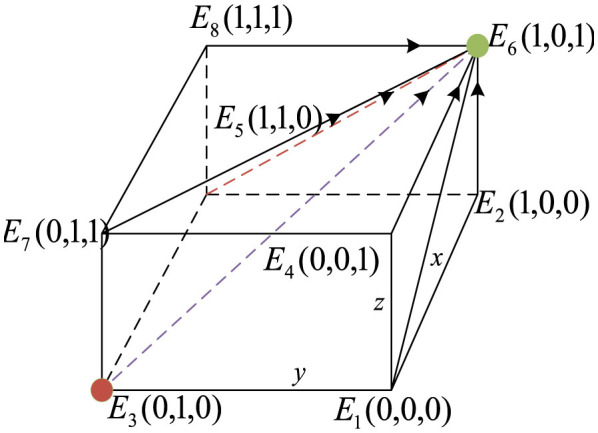
Three-party game phase diagram under scenario 2.

In Table 5, according to Lyapunov stability (38), the stable point is *E*_6_(1, 0, 1), while the unstable point is *E*_3_(0, 1, 0). In this context, medical institutions may choose to engage in fraudulent behavior due to relatively light penalties, while third-party assessment agencies may face heavier penalties under government regulation, shifting from false assessments to truthful assessments. The phase diagram under these conditions is shown in Figure 3.

### 4.3 Scenario 3

When *C*_*j*_ − *C*_*i*_ + *S*_*j*_ < 0, *A*_*j*_ − *C*_*n*_ − *M* + *R*_*i*_ < 0, *A*_*i*_ + *R*_*j*_ − *C*_*m*_ − *B* + *C*_*x*_>0, the *E*_4_(0,0,1) is the evolutionary stable strategy. At this point, the cost to the government of implementing strong regulation exceeds the overall cost of adopting weak regulation, leading the government to ultimately favor weak regulation. The incentive and benefits for medical institutions to provide data truthfully will be less than the benefits to provide false data; therefore, medical institutions will eventually opt to submit false data. Conversely, the benefit to the third-party assessment agencies of conducting a truthful assessment will surpass that of a false assessment, prompting the third-party organization to ultimately choose a truthful assessment. The stability of the equilibrium point is illustrated in Table 6.

**Table 6:** System equilibrium point stability in scenario 3.

Equilibrium point	Eigenvalue			Stability
	#x003BB;_1_	λ_2_	λ_3_	
*E*_1_(0, 0, 0)	< 0	>0	>0	Saddle point
*E*_2_(1, 0, 0)	>0	< 0	>0	Saddle point
*E*_3_(0, 1, 0)	< 0	< 0	>0	Saddle point
*E*_4_(0, 0, 1)	< 0	< 0	< 0	ESS
*E*_5_(1, 1, 0)	>0	>0	>0	Unstable
*E*_6_(1, 0, 1)	>0	>0	< 0	Saddle point
*E*_7_(0, 1, 1)	< 0	>0	< 0	Saddle point
*E*_8_(1, 1, 1)	>0	< 0	< 0	Saddle point

**Figure 4:**
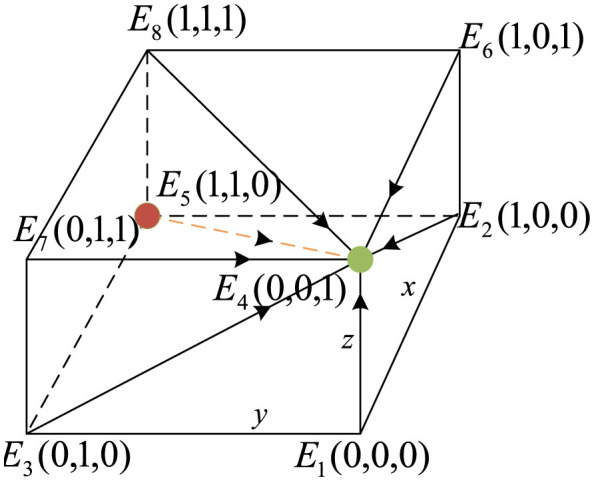
Three-party game phase diagram under scenario 3.

In Table 6, according to Lyapunov stability (38), *E*_4_ (0, 0, 1) is a stable point, while *E*_5_ (1, 1, 0) is an unstable point. In this context, the strategy choices of each subject are analyzed under the condition of significantly high regulatory costs imposed by the government. When regulatory costs are exceedingly high, the government typically opts for a weak regulatory strategy. However, third-party assessment agencies may choose to act truthfully due to concerns about their reputation, whereas medical institutions may resort to dishonesty because of the government's weak regulation. The phase diagram illustrating this condition is shown in Figure 4.

### 4.4 Scenario 4

When *A*_*i*_ + *A*_*j*_ + *C*_*j*_ − *C*_*i*_ + *E* < 0, *C*_*n*_ + *M* − *R*_*i*_ − *A*_*j*_ < 0, *A*_*i*_ + *R*_*j*_ − *C*_*m*_ − *N* + *C*_*x*_>0, the *E*_7_(0, 1, 1) is the evolutionary stable strategy. In this scenario, the cost to the government of implementing strong regulation is higher than the overall cost associated with weak regulation. Consequently, the government's strategy will be to opt for weak regulation. The medical institutions will choose to provide truthful data, as the benefits of doing so outweigh the advantages of submitting false data. Similarly, third-party assessment agencies will opt to deliver truthful assessments because the benefits of truthful assessments surpass those of false ones. The stability of the equilibrium point is illustrated in Table 7.

**Table 7:** System equilibrium point stability in scenario 4.

Equilibrium point	Eigenvalue			Stability
	λ_1_	λ_2_	λ_3_	
*E*_1_(0, 0, 0)	< 0	< 0	>0	Saddle point
*E*_2_(1, 0, 0)	>0	>0	>0	Unstable
*E*_3_(0, 1, 0)	< 0	>0	>0	Saddle point
*E*_4_(0, 0, 1)	< 0	>0	< 0	Saddle point
*E*_5_(1, 1, 0)	>0	< 0	>0	Saddle point
*E*_6_(1, 0, 1)	>0	Uncertain	< 0	Saddle point
*E*_7_(0, 1, 1)	< 0	< 0	< 0	ESS
*E*_8_(1, 1, 1)	>0	Uncertain	< 0	Saddle point

**Figure 5:**
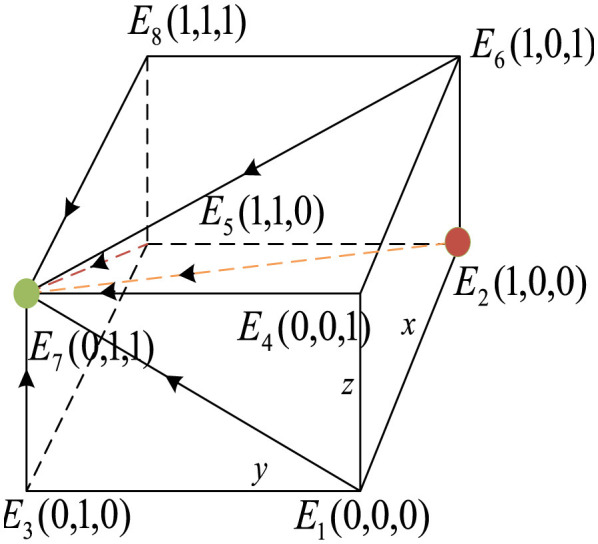
Three-party game phase diagram under scenario 4.

In Table 7, the evolutionary stable equilibrium of the system is *E*_7_(0, 1, 1), where *E*_2_(1, 0, 0) represents an unstable point. In this situation, under strong government supervision, medical institutions and third-party assessment agencies choose to engage in genuine behavior in order to avoid serious punishment. However, when the cost of strong government regulation is too high, the government will choose a weak regulatory strategy. Under weak regulation, if the government rewards real behavior less, the probability of medical institutions and third-party assessment agencies choosing false behavior will increase. Therefore, the final system strategy will shift from *E*_2_(1, 0, 0) to *E*_7_(0, 1, 1). The specific evolutionary phase diagram is shown in Figure 5.

## 5 Numerical simulation

### 5.1 Simulation of phase diagrams

**Figure 6:**
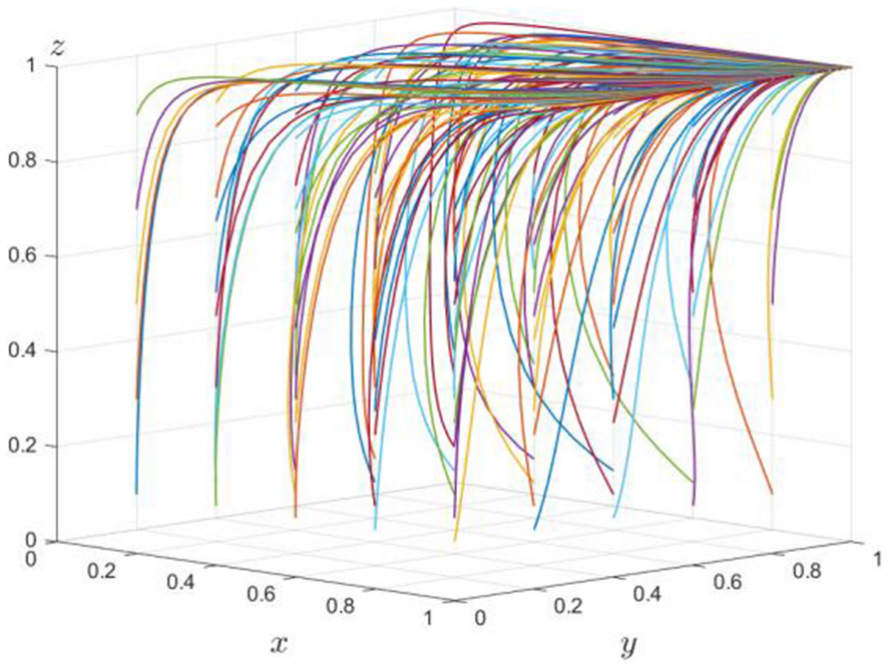
Evolutionary game simulation diagram under scenario 1.

**Figure 7:**
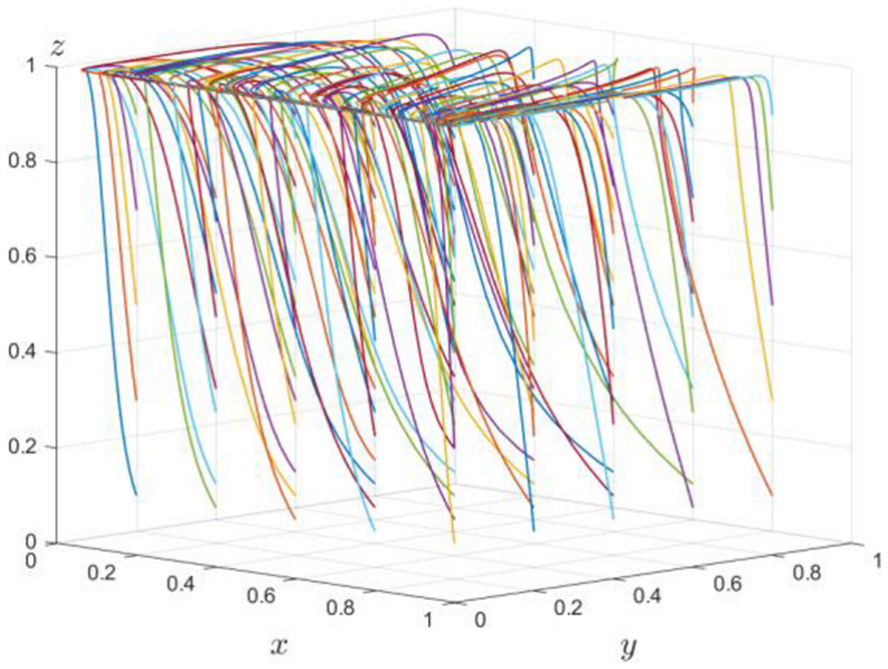
Evolutionary game simulation diagram under scenario 2.

**Figure 8:**
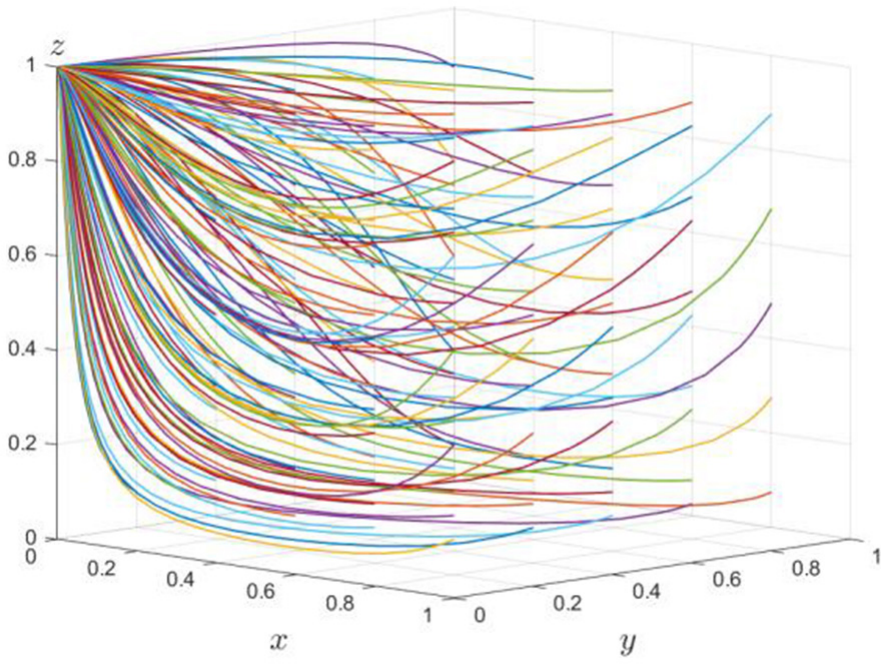
Evolutionary game simulation diagram under scenario 3.

**Figure 9:**
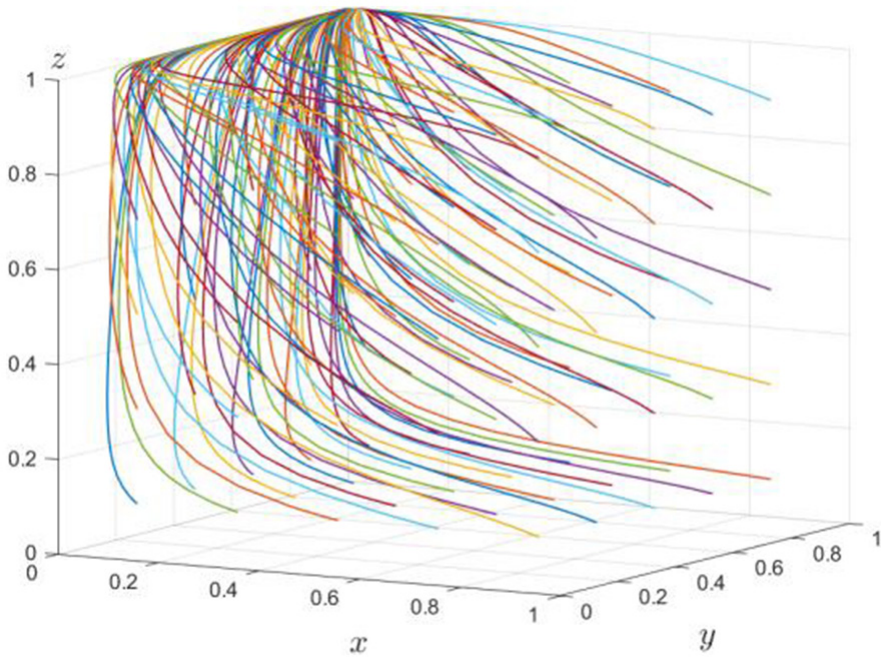
Evolutionary game simulation diagram under scenario 4.

Based on previous research (39, 40), numerical simulation is a good way to verify the correctness of theoretical derivations. This study utilizes MATLAB (2024b) software for numerical simulations to verify the effectiveness of the system evolution stability analysis. The parameter values employed in this study are calibrated based on a combination of legal provisions, regulatory economics principles, and relative ratio relationships, given the limited availability of direct statistical data in this emerging field. Specifically: (1) Penalty parameters (*S*_*i*_, *S*_*j*_, *P*) are set within the range prescribed by the Personal Information Protection Law (enacted in 2021), which stipulates fines of 10,000–100,000 RMB for responsible persons. Accordingly, the fine in this study is set at 10 (i.e., 100,000 RMB); (2) Referring to Qiu et al. (41), regulatory cost parameters (*C*_*i*_, *C*_*j*_) reflect the empirical ratio between intensive and routine enforcement costs in public administration, typically ranging from 3:1 to 5:1. The remaining parameters are calibrated based on the studies of Wang and Ren (42) and Li et al. (28), together with empirical observations from healthcare settings. All parameters are measured in units of 10,000 RMB. This calibration principle is generally applicable, though specific values should be adjusted to reflect scenario-specific conditions. The parameter values for scenarios 1–4 are presented in Table 8, and the simulation results are illustrated in Figures 6–9.

**Table 8:** Parameter assignment.

Scenario	*C_*i*_*	*C_*j*_*	*E*	*F*	*C_*n*_*	*C_*m*_*	*C_*x*_*	*B*	*M*	*N*	*A_*i*_*	*A_*j*_*	*R_*i*_*	*R_*j*_*	*R_*m*_*	*S_*i*_*	*S_*j*_*	*P*
1	10	2	15	20	3	2	1	7	8	5	5	4	12	10	10	8	9	12
2	8	2	10	20	3	2	1	4	13	5	3	2	1	10	9	7	7	5
3	10	3	30	35	3	2	1	7	20	5	6	1	1	10	21	3	2	12
4	58	2	25	30	3	2	1	6	9	4	6	10	8	3	10	6	9	12

As illustrated in Figures 6–9, Scenario 1 ultimately evolves to *E*_8_(1, 1, 1), Scenario 2 evolves to *E*_6_(1, 0, 1), Scenario 3 evolves to *E*_4_(0, 0, 1), and Scenario 4 evolves to *E*_7_(0, 1, 1). This progression is consistent with the theoretical derivation presented earlier.

### 5.2 Simulation of influencing factors

Further comparative analysis of the constraint conditions in the stable scenarios mentioned above reveals several potential factors influencing the strategies of the government, medical institutions, and third-party agencies, specifically: *P*, *A*_*i*_, *S*_*i*_, *S*_*j*_, *R*_*i*_, and *R*_*j*_. The selection of parameters for sensitivity analysis is motivated by their pivotal roles in shaping the strategic interactions among stakeholders. These six parameters encompass the three cor epolicy dimensions in medical data governance. First, the punishment dimension includes *P* (collusion penalty), *S*_*i*_ (penalty on third-party assessment agencies for false assessment), and *S*_*j*_ (penalty on medical institutions for false provision), which capture the government's deterrence mechanisms under strong regulation. Second, the incentive dimension includes *A*_*i*_ (incentive for truthful assessment), reflecting the government's guidance mechanisms under weak regulation. Third, the market return dimension includes *R*_*i*_ and *R*_*j*_, representing the intrinsic economic motivations of medical institutions and third-party assessment agencies, respectively. Together, these parameters constitute the complete set of policy levers available to regulators and the key economic drivers of stakeholder behavior. Therefore, by combining the numerical simulations of Scenario 1, this paper utilizes MATLAB (2024b) for the simulations and conducts an analysis of the influencing factors as follows. The initial strategy proportion for the government, medical institutions, and third-party assessment agencies is set at 0.2.

(1) The effect of *P*

**Figure 10:**
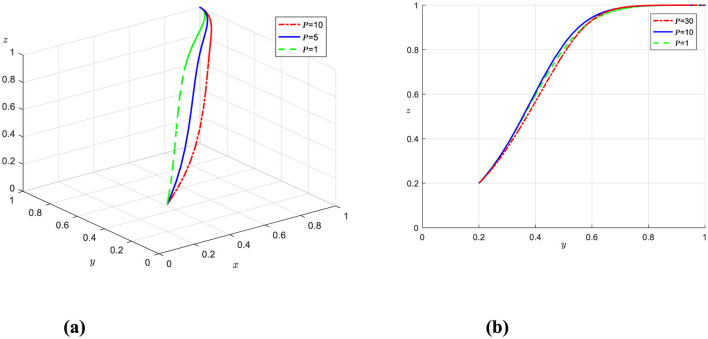
**(a)** Strategy evolution of the medical data management system under different government penalty levels (3D). **(b)** Strategy evolution of the medical data management system under different government penalty levels (*y*–*z* view).

Penalties, as an important means of government regulation, plays a crucial role in medical data management. In order to investigate the impact of government penalties on collusion between medical institutions and third-party assessment agencies, this study increased the penalty *P* from 1 to 10. The impact of government penalty is shown in Figure 10.

As shown in Figure 10, the increase in government penalties has accelerated the convergence of collusion behavior between medical institutions and third-party assessment agencies toward real behavior, and the rate at which medical institutions tend to provide truthful information is faster. The increase in such punishment has also led to the evolution of the government regulatory system from weak regulation to strong regulation, ultimately stabilizing at equilibrium *E*_8_(1, 1, 1). These findings suggest that increasing punishment can crack down on collusive behavior, especially with a greater impact on the behavior of medical institutions.

(2) The effect of *A*_*i*_

**Figure 11:**
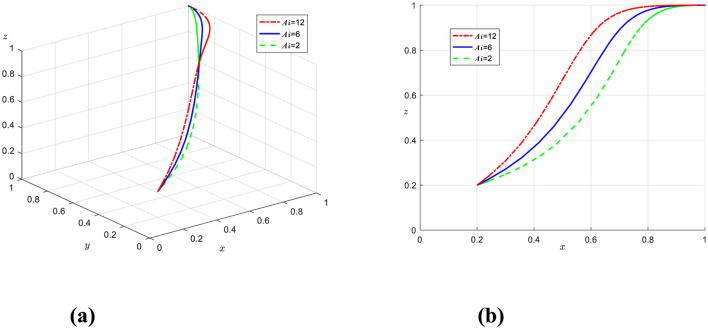
**(a)** Strategy evolution of the data management system under different reward on third-party assessment agencies (3D). **(b)** Strategy evolution of the data management system under different reward on third-party assessment agencies (*x*–*z* view).

Incentives, as a critical component of government governance, play a significant role in data management. This study examines the impact of government incentives on third-party assessment agencies by analyzing how the incentives received by agencies that adopt a truthful strategy under weak government regulation increase from 2 to 12, respectively. The effects of these incentives on the evolution of the system are illustrated in Figure 11.

Figure 11 illustrates the strategic responses of third-party agencies under weak regulatory regimes across varying levels of government rewards. As shown in Figure 11, higher rewards accelerate the convergence of agencies toward truthful strategies. In essence, third-party assessment agencies face a temptation to shirk or collude when short-term gains from dishonesty exceed the benefits of truthful behavior. Therefore, by raising rewards, the government elevates the opportunity cost of dishonesty, making truthful strategies more attractive.

(3) The effect of *R*_*i*_

**Figure 12:**
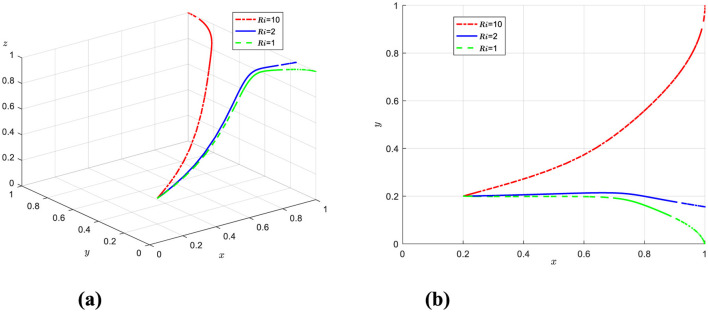
**(a)** Strategy evolution of the system under different return levels of medical institutions (3D). **(b)** Strategy evolution of the system under different return levels of medical institutions (*x*–*y* view).

To examine the impact of truthful strategy benefits on medical institutions behavior, this study establishes that the benefits for real medical institution actions increase from 1 to 10, respectively. The effect of truthful strategy benefits on system evolution results is illustrated in Figure 12.

Figure 12 illustrates the evolutionary paths of game system strategies for medical institutions at varying levels of real revenue. In Scenario 1, as the revenue of medical institutions adopting the truthful strategy increases, the system approaches the state *E*_8_(1, 1, 1) from *E*_6_(1, 0, 1) at an accelerated rate. The critical threshold is derived from the stability condition of *E*_6_(1, 0, 1). Setting λ_2_(*E*_6_) = 0 yields $R_{i}^{*}=C_{n}+M-S_{j}=2$ under Scenario 1 baseline parameters. When *R*_*i*_ < 2, *E*_6_(1, 0, 1) is stable and medical institutions choose false provision; when *R*_*i*_>2, *E*_6_ becomes unstable and the system evolves toward *E*_8_(1, 1, 1). The numerical simulation in Figure 12 confirms this threshold effect, showing that increasing *R*_*i*_ from 1 to 10 drives the system from *E*_6_(1, 0, 1) toward the socially optimal equilibrium *E*_8_(1, 1, 1). This indicates that, although some medical institutions may still opt for deceptive practices when the government enforces strong regulations, an increase in real revenue can influence their strategic choices. In practice, the government can enhance medical institutions' real revenue through specific management techniques to ensure that data management achieves the desired outcomes.

(4) The effect of *R*_*j*_

**Figure 13:**
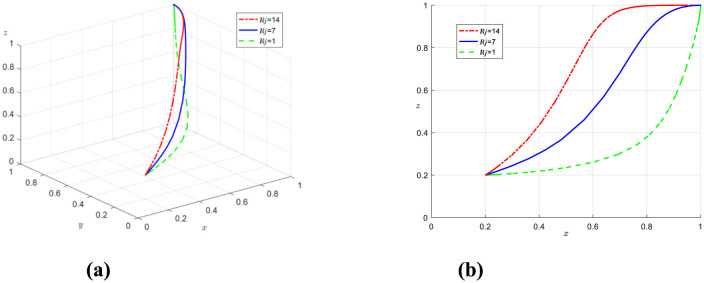
**(a)** Strategy evolution of the system under different return levels of third-party assessment agencies (3D). **(b)** Strategy evolution of the system under different return levels of third-party assessment agencies (*x*–*z* view).

To investigate how the real strategic returns of third-party assessment agencies affect their behavior, this study increased the real strategic returns of third-party assessment agencies from 1 to 14 and observed their impact on system evolution, as shown in Figure 13.

As depicted in Figure 13, the system ultimately stabilizes at equilibrium *E*_8_(1, 1, 1). Notably, as the returns to truthful strategies increase, the convergence speed toward this stable point accelerates. This suggests that higher profitability of honest behavior expedites third-party assessment agencies' adoption of truthful strategies. In practice, when third-party assessment agencies can derive substantial returns from truthful assessments, they are more inclined to cooperate honestly even under stringent oversight. Consequently, enhanced long-term return for truthful agencies can help maintain system stability.

(5) The effect of *S*_*i*_

**Figure 14:**
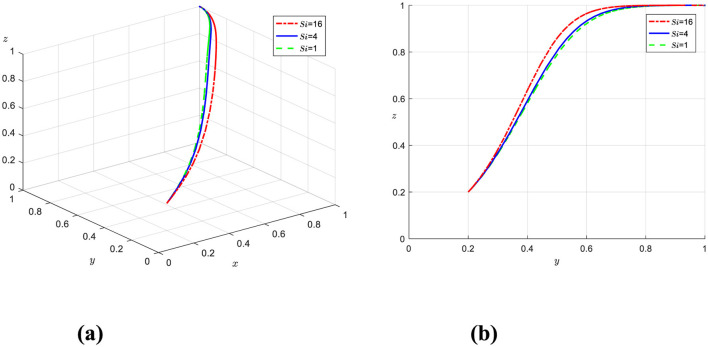
**(a)** Strategy evolution of the data management system under different penalties on third-party assessment agencies (3D). **(b)** Strategy evolution of the data management system under different penalties on third-party assessment agencies (*y*–*z* view).

This study also examines the impact of government penalties on third-party assessment agencies by assigning values ranging from 1 to 16. The effects of these penalties on the system's evolutionary outcomes are illustrated in Figure 14.

As shown in Figure 14, the system ultimately stabilizes at the equilibrium point *E*_8_(1, 1, 1), and the increase in penalty intensity for third-party assessment agencies accelerates the convergence rate of the agency's adoption of truthful strategies. This indicates that the government's punishment of third-party assessment agencies has a significant impact on their strategic decision-making. Therefore, in practice, the government can establish appropriate punishment mechanisms to effectively regulate the behavior of third-party assessment agencies and ensure that they can be truthfully evaluated.

(6) The effect of *S*_*j*_

**Figure 15:**
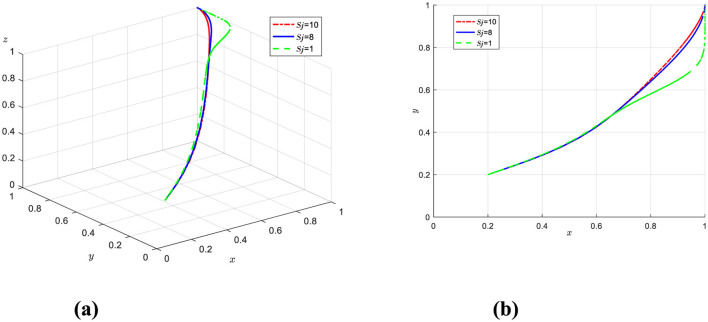
**(a)** Strategy evolution of the data management system under different penalties on medical institutions (3D). **(b)** Strategy evolution of the data management system under different penalties on medical institutions (*x*–*y* view).

To investigate the impact of government penalties on the behavior of medical institutions, this study increased the punishment for medical institutions providing false data from 1 to 10, and examined the influence of government penalties on the strategic evolution of medical institutions, as shown in Figure 15.

Figure 15 illustrates that increasing *S*_*j*_ from 1 to 10 promotes the transition of medical institutions from false to truthful provision, with the system consistently converging to *E*_8_(1, 1, 1). While low punishment levels may temporarily sustain false strategies, the system ultimately reaches the socially optimal equilibrium. The effect of *S*_*j*_ is characterized by gradual convergence acceleration rather than a sharp threshold-triggered equilibrium shift. When the punishment increases, the government's punishment allows medical institutions to quickly transition from false provision to truthful provision. Therefore, increasing the government's punishment will encourage medical institutions to reduce the probability of providing false data, thereby curbing fraudulent behavior by medical institutions.

## 6 Discussion and conclusion

### 6.1 Discussion

This study explores medical data management behavior through evolutionary game theory, focusing on three key participants: the government, medical institutions, and third-party assessment agencies. Through theoretical derivation and numerical simulation, the study clarifies the relationship between medical institution data circulation, agency evaluation, and government regulation. Based on the numerical results presented in Section 5, this section discusses how specific parameters influence stakeholder strategies and system equilibrium.

The simulation results reveal distinct effects of punishment mechanisms, incentive mechanisms, and market return parameters on system evolution. Regarding punishment mechanisms, increasing the collusion penalty *P* accelerates convergence toward the socially optimal equilibrium *E*_8_(1, 1, 1), with medical institutions shifting from false to truthful provision when *P* increases from 1 to 10. This indicates that punishment mechanisms exhibit strong and immediate effects on behavior, with collusion penalties particularly effective in deterring fraudulent behavior. Similarly, penalties on third-party agencies *S*_*i*_ and medical institutions *S*_*j*_ drive rapid convergence to *E*_8_(1, 1, 1). Regarding incentive mechanisms, higher government incentives on third-party assessment agencies *A*_*i*_ accelerates agencies toward truthful assessment, though excessive incentives increase government costs and may shift the strong regulatory strategy. Regarding market returns, higher *R*_*i*_ and *R*_*j*_ enhance the intrinsic motivation for truthful behavior. These findings suggest that governance frameworks should combine severe penalties with market mechanisms that reward data quality.

The comparative analysis of these parameters yields important policy insights. First, punishment mechanisms generally exhibit stronger immediate effects, as evidenced by rapid system convergence under high-penalty conditions. Second, intrinsic market returns play a crucial role in sustaining truthful behavior over the long term, indicating that governance should not rely solely on external regulation. Third, the system is highly sensitive to initial parameter configurations, with strategic interdependencies among stakeholders leading to four distinct equilibrium outcomes. This aligns with conclusions from most evolutionary game studies. The key policy implication is that a balanced combination of severe penalties for collusion, moderate incentives for truthful conduct, and market mechanisms enhancing returns to data quality is most likely to achieve the socially optimal outcome.

### 6.2 Theoretical and practical significance

This study has achieved three specific theoretical breakthroughs. Firstly, by introducing transfer payment *B* and collusion penalty *P* to include collusion behavior between regulated entities, the theory of three-party evolutionary game is expanded, revealing that collusion behavior creates a unique equilibrium path and changes the strategic interdependence among stakeholders. Secondly, this study constructed an asymmetric incentive punishment framework that endogenizes policy choices between incentive and punishment mechanisms based on regulatory intensity, demonstrating that under strong regulation, punishment mechanisms dominate, while under weak regulation, incentive mechanisms are more effective. Thirdly, through Jacobian stability analysis, this study analyzed different equilibrium results and obtained critical thresholds for some variables, which can transform qualitative policy recommendations into quantitative operational guidelines and provide corresponding decision-making insights for relevant decision-making departments.

In practice, this study reveals the dynamic relationships among government entities, medical institutions, and third-party assessment agencies in the realm of medical data management. Firstly, it strengthens policy guidance and regulatory oversight. The government must fully recognize the critical role of medical data management in the era of big data. Therefore, it is essential to enhance relevant policies and regulations, clarify the standards and management requirements for data compliance, and regulate the market order of data-related industries to ensure the long-term stability of data management.

### 6.3 Limitations and prospects

This study discusses the flow and management of medical data in a relevant manner; However, it also has some limitations. Firstly, although this article uses modeling methods to explore the circulation and management of medical data, in practice, medical data management typically involves three or more stakeholders, including patients and other associations. This complexity makes it challenging to fully capture the complexity of the healthcare data management ecosystem. Secondly, although evolutionary game theory can reveal the principles of dynamic strategic evolution, it lacks empirical validation based on actual data, which may affect the generalizability of conclusions. Besides, in terms of modeling, there may be some limitations to the model proposed in this article, such as the possibility of a certain relationship between benefits and costs, which is relatively complex and cannot be expressed using mathematical formulas. In the future, the scope of this study can be expanded to include other stakeholders in the model, allowing for a more comprehensive analysis of the impact of relevant factors on the healthcare data management ecosystem, particularly considering the role of patient participation in healthcare data management. In addition, empirical research methods will be further adopted in the future to investigate which factors affect the flow of medical data and how these factors work.

### 6.4 Conclusion

As a key production factor, the effective circulation of medical data is influenced by the game behavior of multiple stakeholders. This article constructs an evolutionary game model between medical institutions, third-party assessment agencies, and the government. Through theoretical derivation and numerical simulation analysis, it is found that differences in system parameters will lead to four different stable equilibria of evolution. Further research reveals that within a specific range, enhancing the incentive compatibility mechanism and punishment for violations between medical institutions and third-party assessment agencies, improving the benefits of truthful data supply for medical institutions, and suppressing collusion among institutions can help form a fair market trading environment. This will promote the rational pricing and efficient circulation of medical data elements, thereby fully unleashing their potential value.

## Data Availability

The original contributions presented in the study are included in the article/supplementary material, further inquiries can be directed to the corresponding author.
